# HIV-1 residual risk and pre-treatment drug resistance among blood donors: A sentinel surveillance from Gabon

**DOI:** 10.1371/journal.pone.0305935

**Published:** 2024-09-03

**Authors:** Christian Mangala, Désiré Takou, Denis Maulot-Bangola, Grace Beloumou, Olivier Rebienot Pellegrin, Samuel Martin Sosso, Collins Ambe Chenwi, Ezechiel Ngoufack Jagni Semengue, Franck Vigan Codjo, Olga Boussougou, Alex Durand Nka, Michel Tommo, Nadine Fainguem, Rachel Kamgaing, Vicky Ama Moor, Hortense Kamga Gonsu, Veronique Penlap, Thérèse Nkoa, Vittorio Colizzi, Carlo-Federico Perno, Joseph Fokam, Alexis Ndjolo

**Affiliations:** 1 Chantal BIYA International Reference Centre for research on HIV/AIDS Prevention and Management (CIRCB), Yaoundé, Cameroon; 2 School of Health Sciences, Catholic University of Central Africa (ESS-UCAC), Yaoundé, Cameroon; 3 Virology Department, National Public Health Laboratory (NPHL), Libreville, Gabon; 4 Virology Department, National Blood Transfusion Centre (NBTC), Libreville, Gabon; 5 Faculty of Medicine and Surgery, University of Rome Tor Vergata, Rome, Italy; 6 Faculty of Medicine and Biomedical Sciences, University of Yaoundé 1 (FMBS-UY1), Yaoundé, Cameroon; 7 Laboratories of Biochemistry and Microbiology, University Teaching Hospital, Yaoundé, Cameroon; 8 Biotechnology Centre, University of Yaounde I, Yaounde, Cameroon; 9 Faculty of Science and Technology, Evangelic University of Cameroon, Bandjoun, Cameroon; 10 UNESCO Chair of Biotechnology, University of Rome Tor Vergata, Rome, Italy; 11 Department of Microbiology, Bambino Gesu Pediatric Hospital, Rome, Italy; 12 Faculty of Health Sciences, University of Buea (FHS-UB), Buea, Cameroon; 13 National HIV Drug Resistance working group, Ministry of Public Health, Yaounde, Cameroon; Nazarbayev University School of Medicine, PAKISTAN

## Abstract

**Background:**

Surveillance of HIV-1 pre-treatment drug resistance (PDR) is essential for ensuring the success of first-line antiretroviral therapy (ART). Beside population-based surveys, sentinel surveillance of PDR and circulating HIV-1 clades in specific populations such as blood donors could efficiently inform decision-making on ART program. We therefore sought to ascertain HIV-1 residual infection, the threshold of PDR and viral diversity among recently-diagnosed blood donors in Gabon.

**Methods:**

A sentinel surveillance was conducted among 381 consenting blood donors at the National Blood Transfusion Center (NBTC) in Gabon from August 3,2020 to August, 31, 2021. In order to determine the residual risk of HIV transmission, viral load and HIV-1 Sanger-sequencing were performed at the Chantal BIYA International Reference Center (CIRCB)-Cameroon on HIV samples previously tested seronegative with ELISA in Gabon. Phylogeny was performed using MEGA X, PDR threshold>10% was considered high and data were analysed using p≤0.05 for statistical significance.

**Results:**

Five HIV-negative blood donors had a detectable viral load indicating a high residual risk of HIV transmission. Among the samples successfully sequenced, four participants had major drug resistance mutations (DRMs), giving a threshold of PDR of 25% (4/16). By drug class, major DRMs targeting NNRTI (K103N, E138G), NRTIs (L210W) and PI/r (M46L). The most representative viral clades were CRF02_AG and subtype A1. The genetic diversity of HIV-1 had no significant effect on the residual risk in blood transfusion (CRF02_AG, *P = 0*.*3* and Recombinants, *P = 0*.*5*).

**Conclusion:**

This sentinel surveillance indicates a high residual risk of HIV-1 transfusion in Gabon, thereby underscoring the need for optimal screening strategy for blood safety. Moreover, HIV-1 transmission goes with high-risk of PDR, suggesting suboptimal efficacy of ART. Nonetheless, the genetic diversity has limited (if any effect) on the residual risk of infection and PDR in blood donors.

## Introduction

HIV infection still remains a real public health problem. It continues to be prevalent throughout the world, with nearly 39 million people living with HIV (PLHIV), nearly 3/4 of whom are found in Africa. In Gabon, HIV infection is endemic with a prevalence of 3.6% in 2021 **[1–4]**.

The genetic diversity of HIV has a negative impact on the health status of PLHIV. Indeed, this genetic diversity is responsible for several implications in virus behavior, viral pathogenicity, transmission, diagnosis, treatment and vaccine development **[5].**

Molecular epidemiology of HIV is characterized throughout the world by a composite (types, groups, subtypes and recombinants) both in terms of geographical distribution and in terms of the number of HIV-positive people **[6, 7]**. In donors, the molecular epidemiology of HIV is dominated by HIV-1. Studies conducted in Malaysia showed that circulating recombinant forms (CRFs) were significantly prevalent among donors **[8]**. This genetic diversity of HIV-1 plays a role in the persistence of infection worldwide. But controlling this genetic diversity allows for better care of infected people **[9–11]**.

HIV drug resistance can be transmitted when an uninfected person contracts a drug-resistant virus **[12]**. Drug resistance mutations (DRMs) have significantly reduced the success of antiretroviral treatment and, conversely, increased the mortality rate of those infected. Failure to monitor these genotypic DRMs can lead to the transmission of drug-resistant viruses harbored by infected people on treatment to other infected or uninfected people. This is the case of infected naive donors who can transmit their resistant virus to recipients in the event of residual risk **[13–15]**. It is then necessary to carry out genotypic tests to identify the different DRMs to better ensure better care of infected people **[16, 17].** Optimizing the safety of blood donations is becoming a necessity in blood banks around the world in general and in countries with limited resources to ensure a supply of blood bags free of any infectious entity **[18–22]**.

The residual risk (RR) of HIV transmission during blood transfusions constitutes a heavy burden for blood recipients at risk of contracting this virus. Transmission of HIV by transfusion can be avoided in order to spare recipients from this chronic disease which disrupts the habits of any infected patient (economic, dietary, social aspect, etc.). This is why it is important to implement all strategies contributing to the almost total reduction of the RR of HIV in blood transfusion, particularly at the NBTC where the screening algorithm is based on two tests, namely the ELISA test and the chemiluminescence. But despite their simultaneous detection of anti-HIV 1/2 antibodies and the p24 antigen, this screening strategy is not optimal, hence the importance of detecting viral RNA which will ensure optimal transfusion safety **[23, 24].**

In this study, the RR, genetic diversity of HIV-1 and the transmission threshold of genotypic resistance mutations were examined in Gabonese NBTC donors. This study provides the most recent data on the molecular epidemiology of HIV-1 and the transmission of drug resistance mutations in Gabonese blood donors, which could inform the optimal administration of antiretrovirals and improve the screening strategy for HIV in transfusional settings.

## Methods

### Study design and setting

This was a cross-sectional and analytical study was carried out at the National Blood Transfusion Center (NBTC) of Gabon during the period from August 2020 to August 2021. The NBTC is the largest blood bank in Gabon providing its services in terms of labile blood products to almost all health structures of Libreville. And it oversees the application of blood transfusion procedures and the quality of labile blood products in the country. The molecular analyzes were carried out at the Chantal Biya International Reference Center (CBIRC). All participants aged 18–55 years, who donated blood to the NBTC only and who donated blood once during the collection period for residual HIV risk estimation were eligible to participate in the study.

### Ethical considerations

The study was approved by the National Ethics Committee for Research (NECR) of Gabon and by the general management of the National Blood Transfusion Center (NBTC) of Gabon. The number of the ethical opinion certificate was N°0087/2019/PR/SG/NECR. The informed consent form was signed by each study participant.

### Inclusivity in global research

Additional information regarding the ethical, cultural, and scientific considerations specific to inclusivity in global research is included in the S1 Checklist.

### NBTC donation procedure

Blood donations are made at the NBTC by all volunteers and people called compensation or replacement donors. Indeed, all people must meet certain conditions such as being aged 18–70, having a minimum weight of 50kg, absence of chronic illnesses, absence of fasting, no pregnant women, nor women who have given birth less than 6 years ago. months, not having had dental care 2 days preceding the donation, in the absence of recent surgical intervention (in the last 4 months), in the absence of high blood pressure … etc. The blood donation screening strategy at NBTC is based on two serological tests, namely the 4th generation ELISA test detecting anti-HIV-1/2 antibodies and Ag p24. And the chemiluminescence test also detecting anti-HIV-1/2 antibodies and Ag p24. The ELISA test is considered the first test and the chemiluminescence test is considered the second test. All donations that test negative on both tests simultaneously are automatically accepted for distribution. But on the other hand if the donation is positive in both tests or discordant then the blood bag is destroyed.

### Manifest and residual infection

Manifest infection is an infection systematically detectable during screening of blood donations by serological tests, on the other hand, in the case of a residual infection, the donor is declared seronegative but in reality is positive (nucleic acid test). The residual risk (RR) of transmission of a viral infection was calculated by multiplying the incidence rate by the duration of the window period and the whole divided by 365. The duration of the window period is 17 days if the technique used the ELISA and 22 days if the test used is an RDT.

### Serological analysis

Serological analyzes of each donor were carried out using a plasma sample. The techniques used to detect the p24 antigen and anti-HIV-1 (groups M and O) and HIV-2 antibodies in donor plasma were the ELISA technique (Evolis®, BioRad) [25], and the chemiluminescence technique (Cobas® 6000 e601, Roche) [26]. Serological analysis was performed according to the manufacturer’s protocol. The SD Bioline® HIV 1/2 test (Abbott) [27] was used for HIV typing.

### Molecular analysis

#### RNA extraction

Viral RNA was extracted using the Abbott ® viral RNA kit (Abbott m2000, Abbott, USA) [28] which was extracted using the Abbott 2000sp system following the manufacturer’s protocol. An initial reaction mixture of 100μl of magnetic microparticle solution, 2400μl of lysis buffer (lysis buffer + internal control), and 600μl of the sample were contained in a 4.5ml tube and then incubated at 50°C for 20 minutes. The tube was then placed on the red magnetic rack for 2 minutes and then the lysate was removed using a sterile Pasteur pipette. After lysis, 700 μl of wash buffer 1 was added to purify. The contents of the 4.5ml tube were transferred to the 1.5ml tube and the 1.5ml tube was placed on the blue magnetic rack for 1 minute using a sterile Pasteur pipette, the lysate from the tube was removed. Then 700μl of wash buffer 1 was added. After removing the lysate from the tube, 700μl of wash buffer 2 was added to purify by performing two washes. After the last wash, 25μl of elution buffer was added to resurprise the magnetic particles. Incubation of the tube at 70°C for 20 minutes. Then 63μl of wash buffer 2 was added and the tube was placed on the blue magnetic rack for 1 minute. The eluate from the tube was then removed and transferred to another 1.5ml tube. This last tube contains the extracted and purified virus genetic material.

#### Quantification of viral load

The amplification of the cDNA was done using the Abbott ® amplification kit on the Abbott 2000rp system. After obtaining the extract of the genetic material (RNA) of HIV-1, the three amplification reagents were mixed in a 4.5ml tube. Then 200μl of the amplification reagent mixture was dispensed into each well of the microplate. Then 100μl of sample extract was added to each well of the microplate containing the mixture of amplification reagents. The microplate was sealed and then inserted into the thermal cycler to start the cDNA amplification process, the results of which will be given in the form of viral load in 4h30m.

#### Sequencing

A nested PCR was performed to separately amplify the protease (PR) and reverse transcriptase (RT) genes from the cDNA synthesized using the Invitrogen® kit [29, 30]. The reaction mixture consisted of 0.75μl of the BS primer, 0.75μl of the TAK3 primer, 0.8μl of dNTPs, 3μl of MgCl_2_, 5μl of buffer Taq 10x, 33.95μl of H_2_O and 0 .75μl of Taq gold. Then 45μl of master mix was dispensed into the microtube while working on ice. Then 5μl of the sample was distributed in the microtube under the hood. The amplification was done in a thermal cycler. A fragment of amplicons for the PR and RT was generated and confirmed by agarose gel electrophoresis. The purification of the nested-PCR product was done by adding 5μl of enzymes (ExoSAP-IT) in 13μl of the nested-PCR product and it was carried out in the thermal cycler. The sequence reaction used eight primers which completely covered the fragment (approximately 1300 bp) to be sequenced. The reaction mixture was composed of 3.2μl of primers, 1.5μl of big dye, 6.5μl of big dye diluent and 4.8μl of H_2_O. An optical plate was used by distributing in each well 12μl of Formamide HiDi then 7μl of the purified sequence reaction. The denaturation is carried out at 95° C. for 2 minutes. The reading of the optical plate is done using the ABI 3500 Hitachi sequencer. The Recall ® program was used for the interpretation of the chromatogram. Sequences were analyzed in Seqscape and ReCALL and submitted to Stanford HIV drug resistance database, REGA HIV-1 subtyping tool version3.0 and COMET HIV for analysis. The sequences were also analyzed by Calibrated population resistance (CPR) tool from Stanford HIVdb, namely CPR for PRRT sequences.

#### Phylogenetic analysis

The phylogenetic analysis was performed after obtaining the sequences of interest for the study using the sequencing technique. Sequence purification and alignment were done using BioEdit software after downloading the HIV mother sequence (complete genome) and reference sequences of each sequence of interest in the Los Alamos database. MEGA software version 10 was used for the construction of a phylogenetic tree using the Neighbors junction method.

### Statistical analysis

Statistical analysis was performed using SPSS to compare the distribution of mutations in donors with overt and residual infection. And also, to show whether the genetic diversity of HIV-1 had a significant effect on the residual risk in blood transfusion. Donor demographics were expressed as frequencies and percentages.

## Results

### Sociodemographic data of blood donors

Of 381 blood donors who participated in the study, men were more representative than women, respectively 66.9% and 33.1%. The most representative age group was between 25–34 years old (58.0%). Family donors and regular/former donations were more representative with 60.1% and 60.4% respectively (Table 1).

**Table 1:** Sociodemographic data of blood donors, N = 381.

Variables	N	%
**Sex** Male	255	66.9
Female	126	33.1
**Age group**		
18–24	62	16.3
25–34	221	58.0
35–44	74	19.4
45–55	24	6.3
**Donation status**		
Former/Regular	230	60.4
New	151	39.6
**Type of donor**		
Unrelated volunteer	152	39.9
Family	229	60.1

**N:** Number; **%:** Percentage

### Risk of HIV transmission

The study population consisted of 22 HIV-positive and 359 HIV-negative donors. Five HIV-negative donors had detectable viral load. The RR was high in transfusion settings with 64.8 per 100 thousand donations **[31].**

### Characterization and phylogenetic analysis of HIV-1 strains

**Fig 1:**
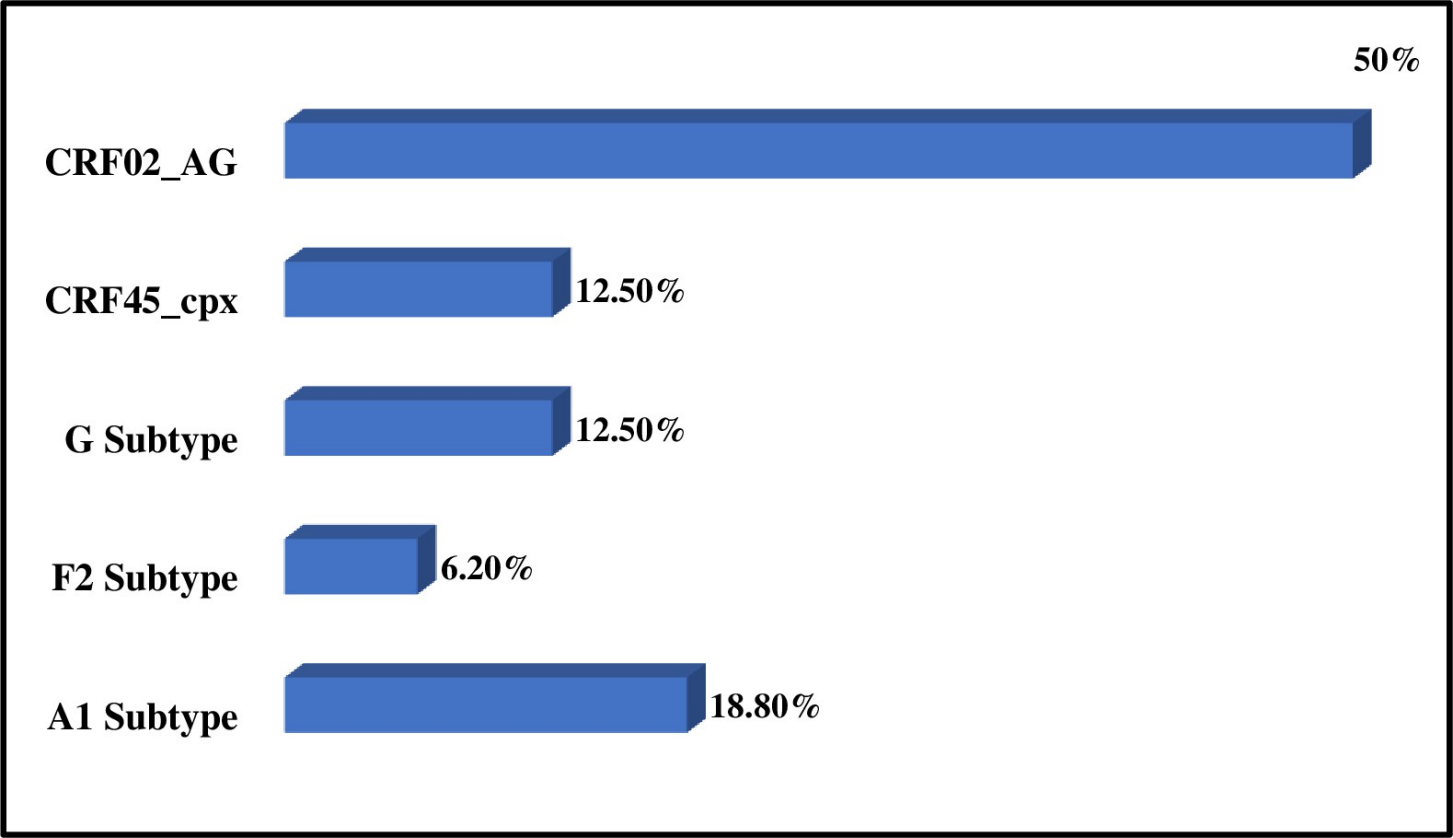
Characterization of molecular strains of HIV-1 in donors. A1, F2, G: HIV-1 group M subtypes. CRF45_cpx and CRF02_AG: Recombinant forms of HIV-1 group M.

**Fig 2:**
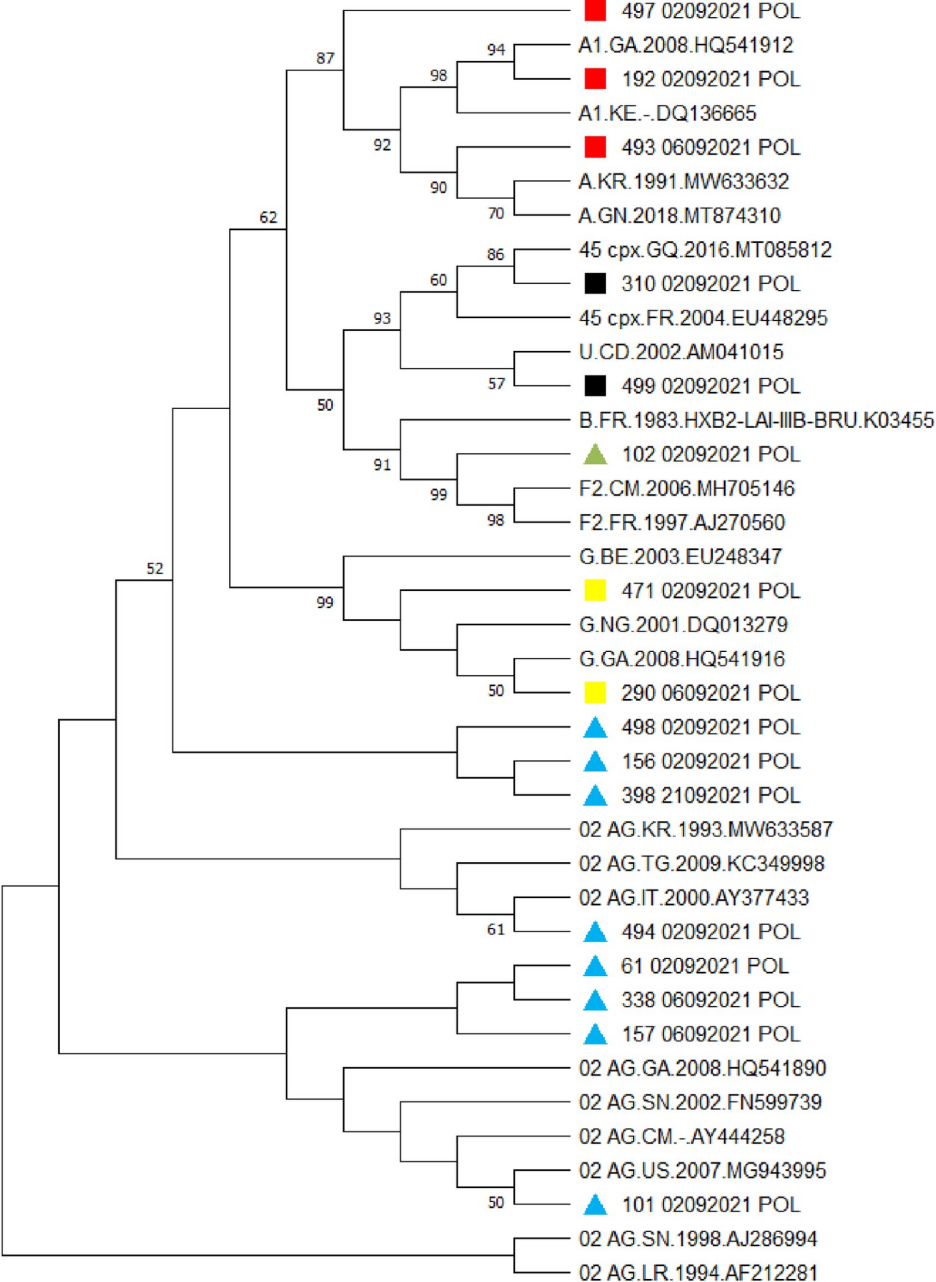
Phylogenetic tree of HIV-I strains. On the phylogenetic tree, strains Al, F2, G, CRF02_AG and CRF45_cpx were identified with colored symbols in red, green, yellow, blue and black respectively. Reference strains downloaded from the Los Alamos database. Out of six clusters identified, three clusters had drug resistant strains. These six clusters constituted of the sequences identified in Gabon, Equatorial Guinea, Italy, Democratic Republic of Congo and the United States. The percentage of replicated trees in which related strains are clustered in the bootstrap test (500 replicates) is shown next to the branches.

The most representative HIV-1 viral clades in the study donors came from the 16 strains sequenced. Of the 16 strains sequenced, 2 came from HIV-negative donors and 14 came from HIV-positive donors. Among which, they were subtype A1, subtype G, subtype F2, CRF02_AG and CRF45_cpx **[31**] (Fig 1). The molecular evolutionary genetic analysis tool (MEGA X) made it possible to carry out the phylogenetic analysis of the RT and PR sequences. There was an evolutionary relationship between the study strains and those available in the Los Alamos database. On the phylogenetic tree, the study strains A1, F2, G, CRF02_AG, and CRF45_cpx were identified with symbols colored in red, green, yellow, blue, and black respectively. Six clusters were identified and grouped with A1, G, CRF02_AG and CRF45_cpx subtypes; Out of six clusters identified, three clusters had drug resistant strains. These six clusters constituted of the sequences identified in Gabon, Equatorial Guinea, Italy, Democratic Republic of Congo and the United States (Fig 2).

### Effect of HIV-1 genetic diversity on residual risk in transfusion

The determination of the viral load and the genotyping of the different molecular strains made it possible to show whether genetic diversity affected the residual risk in blood transfusion. And to show that the genetic diversity of HIV-1 affected the residual risk, it was necessary to compare the strains isolated in the two categories of infections (manifest and residual). The residual case was part of the CRF02_AG (recombinant) strains. The Fisher exact test had been used to show the association between residual infection and CRF02_AG strains and non-CRF02_AG strains on the one hand, and on the other hand between residual infection and sub-types and recombinants. Statistical analysis revealed that HIV-1 genetic diversity had a limited effect on residual risk (with p-value varying between 0.30 to 0.50) (Table 2).

**Table 2:** Effect of HIV-1 genetic diversity as a function of CRF02_AG, non-CRF02_AG, subtypes and recombinants.

		Viral strains				Viral strains			
		CRF02_AG	No CRF02_AG	Total	*P-value*	Subtypes	Recombinants	Total	*P-value*
**Residual Infection**	Absence	7	8	15	*0*.*30*	6	9	15	*0*.*50*
	Presence	**1 (12.5%)**	**0 (0%)**	**1**		**0 (0%)**	**1 (10%)**	**1**	
	Total	8	8	16		6	10	16	

### Prevalence of drug resistance mutations in donors

Major drug resistance mutations (DRM) were identified based on Stanford’s interpretation algorithm and standardized lists of transmitted mutations (https://hivdb.stanford.edu/dr-summary/mut-scores/NRTI/). HIV-1 strains with genotypic mutations associated with major drug resistance were identified in 4 cases (25%) out of 16 strains from naïve donors. Of the 4 resistant strains, there was an A1, G subtype, and two CRF02_AG recombinants. The major resistance mutations identified consisted of K103N, E138G, L210W, and M46L. Inheritance of the K103N resistance mutation only inhibits the antiretroviral activity of efavirenz and nevirapine among all non-nucleoside reverse transcriptase inhibitors (NNRTIs). But the transmission of the E138G mutation only slows down the antiretroviral activity of NNRTIs except for doravirine. The L210W resistance mutation interferes with the antiretroviral activity of nucleoside reverse transcriptase inhibitors (NRTIs) except for emtricitabine and lamivudine. And finally, the M46L resistance mutation slows down the antiretroviral activity of protease inhibitors (PIs) except for darunavir/r. The overall threshold for transmission of resistance mutations was 25% [95% CI: 10; 50]. New donors and family donors had thresholds for the transmission of resistance mutations respectively 28.6% [95% CI: 12; 55] and 25% [95% CI: 9; 53] (Table 3).

**Table 3:** HIV-1 resistance mutation transmission threshold according to donor profile.

Variable		Mutations	N. of strains	Transmission threshold (%), 95% CI	*P-value*
		*K103N*, *E138G*, *M46L*, *L210W*			
	All	4	16	25 [10–50]	
**Sex**	Male	3	10	30 [11–60]	*0*.*50*
	Female	1	6	16.7 [3–56]	
**Age (years)**	18–24	0	1	0	*0*.*20*
	25–34	2	11	18.2 [5–48]	
	35–44	2	4	50 [15–85]	
	45–55	0	0	0	
**Donation status**	New	4	14	28.6 [12–55]	*0*.*50*
	Former/Regular	0	2	0	
**Type of donors**	Family	3	12	25 [9–53]	*0*.*50*
	Volunteer	1	4	25 [4–70]	

**N:** Number; **%:** Percentage; **95% CI:** 95% Confidence interval

## Discussion

The risk of HIV-1 transmission in transfusion settings and drug resistance remain a current problem in all developing countries. In this study, the objective was to ascertain HIV-1 residual infection, the threshold of PDR and viral diversity among recently-diagnosed blood donors in Gabon. In this study, the distribution of blood donors according to gender was in favor of men with 66.9%, and according to age group, it was in favor of the age group between 25–34 years with 58.0%. The sex ratio (Male/Female) was 2:1. This could be explained by the fact that the blood donors most motivated to give blood in African blood banks in general and in Gabon in particular, are men. But there would also be the woman’s physiology (pregnancy, menstruation, etc.) which would prevent women from donating more blood. In addition, the low representation of older blood donors could be explained by the high rate of chronic conditions (diabetes, high blood pressure, cardiovascular diseases, etc.) encountered in this age group (45–55 years). Similar results were observed in studies conducted in Cameroon, Central African Republic (CAR), Ghana and Pakistan [**32–35**]. The residual risk of HIV-1 transmission was estimated at 64.8 per 100 thousand donations **[31]**. These results showed that the residual risk of HIV-1 at the Libreville NBTC was high compared to that estimated in 2014 which was 64.7 per 1 million donations. And this could be explained by an ineffective screening strategy despite the use of 4th generation screening tests which are sometimes not subject to local evaluation or by the absence of tests detecting viral nucleic acids (RNA or DNA) in plasma. Some studies carried out in Cameroon, Ghana, Burkina Faso and Tanzania showed that the residual risk of HIV-1 was present and high in several blood banks in resource-limited countries **[36–41]**. This could be justified by certain activities such as tourism and travel which promote the circulation of strains from one country to another, from one continent to another. Some African authors showed that CRF02_AG and A1 subtypes were more representative **[1, 2, 31, 42, 43]**. Phylogenetic analysis identified six sequences clustered with subtypes A1, G, CRF02_AG, and CRF45_cpx. Of the identified groups, three consisted of HIV-1 subtypes with drug resistance mutations. This could be explained by the modification of the molecular map of HIV-1 strains due to socio-epidemiological factors, namely migration and mobility of populations from one country to another. These corroborate with those obtained in several studies **[2, 8, 44, 45]**. Statistical analysis showed that the genetic diversity of HIV-1 had a limited (or even non-existent) effect on the residual risk of infection in the transfusion context. The threshold for transmission of resistance mutations from one individual to another is a major issue in blood banks. The study showed that the threshold for transmission of major resistance mutations was 25% in donors compared to critical threshold of 10% set by the WHO. But despite the small size of the strains sequenced, this could mean that one in four donors would carry an HIV-1 strain with a major resistance mutation that could transmit a resistant virus to a recipient in the event of residual risk. New male family donors aged 35 to 44 years had a high frequency (50%) of possession of an HIV-1 strain with a major resistance mutation. Indeed, the mutations identified during the study have a negative impact on the antiretroviral activity of certain antiretroviral molecules such as Efavirenz and Nevirapine. But it is important to specify that these strains already possessed this major resistance mutation (K103N) before their transmission to infected donors. In some countries these data have been observed on the transmission of drug resistance mutations (DRM) in positive donors **[17, 46–48]**. The L210W mutation interferes with the antiretroviral activity of nucleoside reverse transcriptase inhibitors (NRTIs), including zidovudine, abacavir and tenofovir. Some authors showed that L210W had a negative impact on zidovudine activity in combination with other mutations **[9, 46, 49–51]**. The M46L mutation is a major resistance mutation selected by protease inhibitors (PIs) **[13, 15, 43]**. Detection of DRMs in infected people will help prevent and reduce drug resistance. Some limitations of the study were observed. Given the low rate of HIV-1 positivity in our study population, few samples were eligible for genetic sequencing for the assessment of genetic diversity, which would limit the control of viral strains circulating in Gabon.

Depending on the sequencing technique used (Sanger method), the detection of viral genotypes/strains would be limited to the majority populations (covering at least 20% of the viral population present in the individual concerned). This gives rise to future studies with high-throughput sequencing analyzes (up to 1% coverage of the viral population in the individual concerned).

## Conclusion

The risk of viral transmission through blood transfusion persists in blood banks. Despite the significant circulation of HIV-1 strains, they have no significant effect on the residual risk in blood transfusion. The risk of transmission of DRM exceeds the critical threshold of 10% set by the WHO. This constitutes a real concern for infected donors likely to transmit resistant viruses to recipients. It then becomes necessary to strengthen transfusion safety by detecting the viral genome and sorting donors. In addition, the implementation of the genotypic resistance test for the care of PLHIV in Gabon could become a necessity.
